# Impact of variation in voluntary moderate deep inspiration breath hold on the 3D dose distribution in breast cancer radiotherapy

**DOI:** 10.1016/j.tipsro.2026.100385

**Published:** 2026-02-19

**Authors:** Lotte van der Werf, Lars Murrer, Karolien Verhoeven, Debby Knarren, Kirsten Kremer, Femke Vaassen, Liesbeth Boersma, Michel Öllers

**Affiliations:** Department of Radiation Oncology (MAASTRO), GROW Research Institute for Oncology and Reproduction, Maastricht University Medical Centre, Maastricht, Netherlands

**Keywords:** SGRT, Breast cancer, Breath hold, Organs at risk

## Abstract

**Aim:**

Determination of impact of variations during voluntary moderately deep inspiration breath hold (vmDIBH) on doses to organs at risk (OAR) and target coverage in patients undergoing radiotherapy (RT) for left-sided breast cancer (BC).

**Methods:**

We recorded vmDIBH-breathing signals using a Surface Guidance system, without visual coaching, in 21 BC patients. In 13 patients, cone-beam CT (CBCT) scans were acquired with vmDIBH, immediately after treatment delivery. A 3D dose recalculation was performed on CBCT, cranio-caudally stitched with the planning CT for full dose evaluation. Mean Heart Dose (MHD), Mean Lung Dose (MLD) and V_95%_ of the clinical target volume (CTV) were calculated. Relationships between vmDIBH level, stability and differences in Dose-Volume Histogram (DVH) parameters were analysed using linear regression and Mann-Whitney U-tests.

**Results:**

Of 150 recorded vmDIBHs, 39% of the breath-holds were within a predefined 3 mm gating window. The average deviation from this window was 0.6 mm (SD 1.9 mm), with the mean value just outside predefined boundaries. All recalculated CBCTs (n = 95) met predefined DVH criteria for MHD, MLD, and V95% of tumourbed CTV. The V_95%_ of the whole breast CTV showed minor deviations from the planned dose (mean delta V_95%_: −0.8%, SD 1.6%). For patients with excursions beyond the 3 mm window, average differences were 1.43% in V_95%_-CTV, 0.02 Gy in MHD, and 0.05 Gy in MLD.

**Conclusion:**

Only small breathing excursions beyond 3 mm were observed during vmDIBH without visual coaching, not resulting in clinically relevant CTV Underdosage with MHD and MLD remaining within tolerance.

## Highlights

• Variations were assessed using SGRT vmDIBH without visual coaching in 21 patients.
• 61% of BHs exceeded a 3 mm gating window, but dose deviations were minimal.
• All mean heart and lung doses remained within clinical limits.
• Findings support the feasibility of vmDIBH without continuous visual feedback.

## Introduction

Adjuvant radiation treatment (RT) of the breast or chest wall reduces the risk of breast cancer recurrence with a factor 3–4 [1]. However, in case of left-sided treatment, it has also been reported to yield cardiac injury, leading to an increase in acute coronary events (ACE) [[2], [3], [4]]. Cardiac dose remains clinically relevant, as established by large cohort and meta‑analytic evidence [5,6]. Since the risk on radiation-induced cardiac morbidity is related to the dose received by the heart [[3], [4], [5], [6]], many efforts have been made to reduce the dose to the heart by increasing the distance between the heart and the target volume using a breath hold (BH) technique. Performing a BH has indeed shown to reduce the mean heart dose (MHD) considerably up to 3.4 Gy compared to free breathing [7,8]. Although the BH reduces the MHD, the reproducibility of this BH during an RT series is of importance. Several studies have been performed to investigate both the intra- and inter-fraction reproducibility of the BH. In these studies, methods to improve reproducibility varied from just a short training/practice session or applying the BH without any further assistance (voluntary moderate deep inspiration BH (vmDIBH)) [9,10], to a completely controlled BH using an ABC (active breathing control) device [[11], [12], [13]], or surface guided RT (SGRT) with or without visual coaching [[14], [15], [16], [17], [18]] or advanced mechanical ventilation techniques supported by SGRT [13]. Here, ‘moderate’ denotes a submaximal, maintainable inspiration.

When performing a vmDIBH with SGRT guidance, the patient is monitored during beam-on of the linac by the SGRT cameras. For most linacs nowadays, an automated beam on or off trigger can be initiated, where a beam-hold is automatically initiated when the respiration signal exceeds the tolerances of the predefined breathing window. SGRT improves setup accuracy using external surface matching, and enables intra/inter‑fraction monitoring in breast RT [[19], [20], [21], [22], [23]]. Prior DIBH reproducibility studies relied on SGRT visual coaching and/or beam gating, thus analysing motion only within the gating window [[14], [15], [16],[24], [25], [26]]. In contrast, we evaluate vmDIBH without visual feedback and quantify the dosimetric impact of deviations outside the gating window via CBCT based 3D dose recalculation [27].

In our department, the question arose whether we should add SGRT to our standard patient set-up for our left-sided breast cancer patients, where standard orthogonal kV-kV imaging and/or cone-beam CTs (CBCTs) are used to match on bony anatomy in combination with surgical clips [10]. As SGRT systems are currently implemented only on a limited number of treatment machines in our clinic divided over two physical locations, a large-scale introduction of SGRT for all (left-sided) breast cancer patients including visual coaching and feedback, introduced some logistical challenges. Consequently, we wanted to gain insight into the clinical benefit of adding SGRT to our standard treatment. Therefore, as a first step, the aim of this study was to investigate how well our breast cancer patients were able to perform a vmDIBH by monitoring their vmDIBH using our SGRT system. The stability and variation in vmDIBH during treatment was investigated and the impact on dose deviations to the organs at risk (OARs) and CTV target coverage was assessed.

## Materials and methods

### Study design and patient population

This prospective single-institution cohort study included consecutive patients between August and November 2022. Eligible patients were those receiving radiotherapy to the left breast and able to perform a vmDIBH, as assessed during the planning CT scan. Breathing signals were recorded during treatment using a surface guidance system (C-RAD AB, Uppsala, Sweden). Cone-beam CT (CBCT) scans were planned for evaluation of dose delivery accuracy.

### Fractionation, treatment preparation and technique

**Fig. 1:**
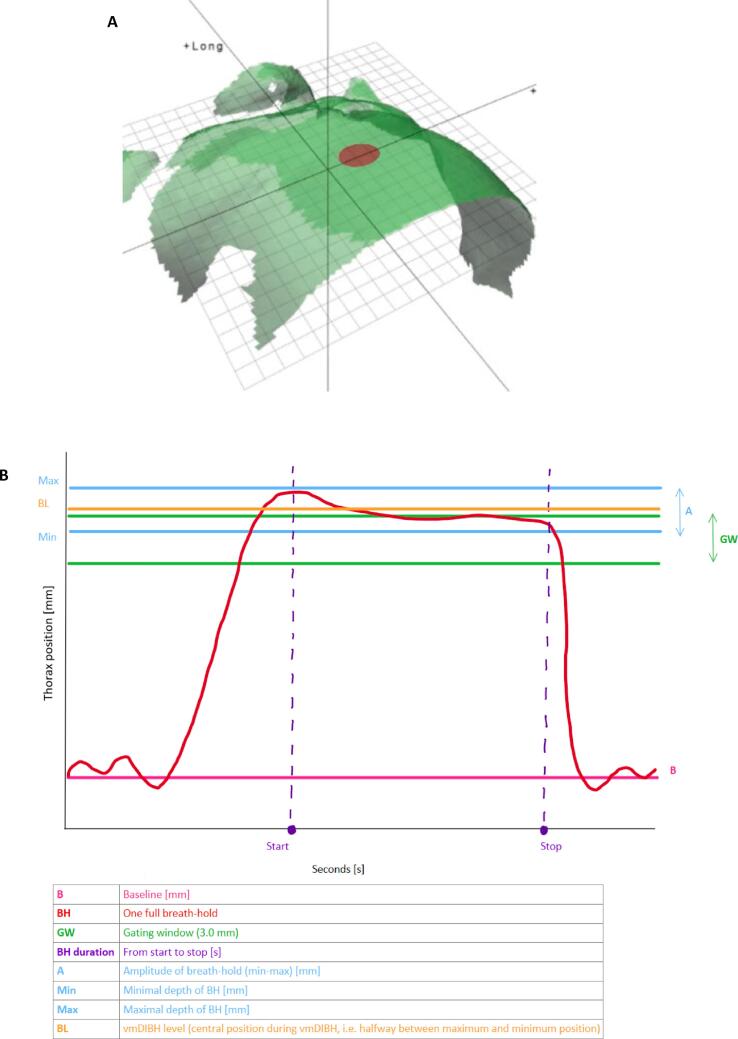
Point of measurement for surface monitoring (A), and a typical breathing pattern as recorded by surface guidance (B). Indicated are: baseline level (B), one full breath-hold (BH), Amplitude (A), minimal and maximum depth of the breath-hold (Min and Max), and optimal window of breath-hold (OW) (width 3 mm). Example of body contour of surface guidance system. Red dot indicates the location of the reference (xyphoid). The recorded breath-hold meets the 3.0 mm stability criteria but exceeds the preset window because of deeper breathing (difference in breathing level, Thorax position [mm]). Measured over time (seconds [s]). In this case, the vmDIBH level is outside the gating window (For interpretation of the references to colour in this figure legend, the reader is referred to the web version of this article).

Fractionation schedules followed departmental protocols (5–20 fractions, depending on boost indication). Patients practiced vmDIBH (20–30 s) briefly before CT acquisition. The planning CT was performed in vmDIBH, using a Siemens Somatom Drive CT in combination with surface scanning for CT (Sentinel, C-RAD AB, Uppsala, Sweden). During the CT scan the excursion from the baseline in free breathing to the vmDIBH level centered in the gating window was determined as a reference level (Fig. 1B, centre of the gating window). The gating window was set to 3 mm (vmDIBH level +/- 1.5 mm) based on clinical experience and supported by other groups [28].

The Clinical Target Volume (CTV) of the breast (CTV1) and tumour bed (CTV2) were delineated according to the ESTRO guidelines [29] and Boersma et al. [30], respectively. The Planning Target Volume (PTV) was derived by expanding the CTV with 5 mm and cropping this structure with 5 mm below the skin. Subsequently, a treatment plan was designed using 6 MV photons using the Eclipse Treatment Planning System (version 16, Varian Medical Systems, Palo Alto, USA) and final dose calculations were performed using the Acuros XB dose calculation engine reporting dose-to-medium on a 2.5 mm dose grid.

Following our internal planning guidelines, a hybrid treatment technique was chosen consisting of 2 tangential fields (2 cm flash margin), with the addition of one or more arcs. The dose distribution had to fulfil several specifications (see Table 1A) [31,32].

**Table 1:** Constraints used for the 3D dose distribution, based on Hurkmans et al. [31] and Brunt et al [32] (Table 1A). Table 1B shows the dose volume parameters determined from the dose-distribution of the planning CT and of the recalculated dose distribution on the stitched CBCTs. CTV = clinical target volume; PTV = Planning Target Volume, CL = contralateral, MHD = Mean Heart Dose; MLD = Mean Lung Dose.

PTV coverage and OARs	Constraints
PTV coverage	V_95%_-PTV ≥ 98% and mean PTV Dose 99–101%
Heart constraints in case of 15 or 20 fractions	MHD < 2 Gy or MHD < 3 Gy
Lung constraints in case of 15 or 20 fractions	MLD < 3 Gy or MLD < 5 Gy
Heart constraints in case of 5 fractions	MHD < 1.3 Gy and V_1.5Gyheart_ < 30% and V_5Gyheart_ < 10% and V_7Gyheart_ < 5%
Lung constraints in case of 5 fractions	MLD <1.9 Gy and V_8GyLung_ < 15% (ipsilat lung)

**Parameter**	**Definition**
V_95_ − CTV1	% of volume of the CTV1 receiving at least 95% of the prescribed dose
V_105_-CTV1	% of volume of the CTV1 receiving at least 105% of the prescribed dose
V_107_-CTV1	% of volume of the CTV1 receiving at least 107% of the prescribed dose
V_95_ – CTV2*	% of volume of the CTV2 receiving at least 95% of the prescribed dose
V_105_-CTV2*	% of volume of the CTV2 receiving at least 105% of the prescribed dose
V_107_-CTV2*	% of volume of the CTV2 receiving at least 107% of the prescribed dose
D_mean_-PTV1	Mean dose in PTV1 in Gy and % of prescribed dose
D_max_-PTV1	Maximum dose in PTV1 in Gy and % of prescribed dose (vol > 1,8 cc)
D_mean_-PTV2*	Mean dose in PTV1 in Gy and % of prescribed dose
D_max_-PTV2*	Maximum dose in PTV2 in Gy and % of prescribed dose (vol > 1,8 cc)
MHD	Mean dose in the heart in Gy
MLD	Mean dose over both lungs in Gy
D_mean_- CL-breast	Mean dose in the contralateral breast in Gy

* = only CTV2 and PTV2 available for patients with a boost to the tumour bed.

### Treatment and set-up monitoring

Patients were treated on a Truebeam linac (Varian Medical Systems, Palo Alto, USA). In case of a 5-fraction scheme, CBCT was used for set-up for all fractions, and was matched with the planning CT based on the bony anatomy (thoracic wall and sternum), soft tissue (breast and skin), and surgical clips. For patients that were treated with 15 fractions or more, treatment set-up was performed with AP and lateral kV-kV imaging, using the surgical clips to match on [10]. CBCTs in vmDIBH were made after the first 3 fractions and in the further scheme CBCTs were only made when set-up and match tolerances were violated based on the kV-kV/DRR images. Treatment was interrupted only if the patient was repeatedly unable to maintain a vmDIBH for the duration of the treatment beam being delivered (approximately 20–30 s).

### Additional study procedures

For the current study, surface scanning with simultaneous monitoring of the breathing signal was performed during treatment. The primary respiratory gating point, a circular area that measured the vertical position of all surface points within that point, was the xyphoid (Fig. 1A). During linac set-up, patients were asked to perform a vmDIBH without visual coaching. In at least 3 fractions, an additional CBCT in vmDIBH was performed immediately after delivery of the fraction. The breathing signal was recorded during the CBCT after the fraction without shifting the patient (patient in position of pre-irradiation set-up procedure). Since for technical reasons we could not store the breathing signal, video screen captures were made within the 4D software that controls the Catalyst system (C-RAD AB, Uppsala, Sweden). For subsequent analysis, video screen captures were obtained during the additional CBCT procedures.

The CBCTs were used for dose recalculation also in the Eclipse Treatment Planning System (version 16, Varian Medical Systems, Palo Alto, USA). Due to the limited Field of View (FOV) of the CBCT, the missing information of the CBCT was completed by cranio-caudally “stitching” the planning CT to the CBCT, based on the clinical registration of the CBCT and the planning CT, to allow calculation of the mean lung dose (MLD).

### Data extraction and recording

Breathing motion was quantified by measuring the *amplitude* during vmDIBH (difference between the minimum breathing position (lowest excursion during vmDIBH) and the maximum breathing position (highest excursion during vmDIBH)) and the *breathing level* (central position during vmDIBH, i.e. halfway between maximum and minimum position) from the obtained videos (Fig. 1B). This was done both during set-up and during the CBCT immediately after delivery of the fraction.

For the CBCT performed after the fraction, we also recorded the *duration* of the vmDIBH, as shown in Fig. 1B. The start of the breath-hold was defined as the point at which inhalation flattened, and the stop at the initiation of full exhalation. Furthermore, we scored whether the patient *maintained the vmDIBH* within a 3 mm window centered around the breathing level determined on planning CT, based on a previous study [33].

The CT and CBCT data were anonymized and stored in the treatment planning system (Aria Eclipse version 16.0, Varian Medical Systems, California, USA). Several dose-volume histogram (DVH) parameters for the CTVs and for the heart, lung and contralateral breast were automatically extracted using an in-house developed script, for both the planning CT and the recalculated dose distributions on the stitched CBCTs (Table 1B).

### Statistical analysis

Descriptive statistics were applied to evaluate patterns in the breathing signal and DVH parameters. A significance level of p < 0.05 was considered statistically significant for all tests. Differences between recalculated CBCT-based dose metrics and planning CT values were assessed using one-sample t-tests.

Delta values were computed by comparing the recalculated dose distributions on the stitched CBCTs with those from the planning CT, and by using the clinical planning criteria for target volumes and organs at risk. Specifications of the PTV coverage (V_95%_ > 98%) were considered to be met if the V_95%_ of the CTV > 99% in the recalculated dose distribution on the stitched CBCT [34].

To assess whether these delta values were correlated to differences in breathing level between set-up and post-treatment CBCT, linear regression was applied. Additionally, we compared delta values between patients who maintained their vmDIBH within the predefined 3 mm gating window and those who deviated from it (either above or below). Mann-Whitney U-tests were used to test for statistical significance.

The baseline breathing level on CT was defined from the CRAD software. During treatment set-up, a new baseline (purple line, Fig. 1B) was acquired on the linac prior to CBCT acquisition, which served as a reference for the definition of the gating window during treatment. The gating window was superimposed on the new baseline for every single fraction.

### Ethical consideration

Patient consent was waived by the Ethics Committee (W 22 10 00059) in accordance with national regulations, as the study involved no additional interventions or patient burden beyond standard care. All data were anonymized prior to analysis and handled according to institutional standards. The study posed no additional risk to patients, and treatment delivery was not influenced by study procedures.

## Results

### Patient characteristics and available data

A total of 21 patients were included in this study with a median age of 65 years (range 39–82, mean 64.9). vmDIBH breathing signals were recorded during treatment, resulting in 150 individual vmDIBH manoeuvres: 55 during the initial pre-treatment set-up and 95 during CBCT acquisition immediately after treatment delivery.

Of the 21 included patients, 13 had complete CBCT data suitable for dose recalculation, resulting in 36 CBCT scans. Patients without CBCT data were excluded from the dosimetric analysis but were still included in the descriptive analysis of vmDIBH stability. Missing CBCTs were due to technical limitations rather than patient-related factors, minimizing the risk of selection bias. Of these 13 patients: three received a simultaneous integrated boost (SIB) of 20 × 2.18 Gy to the breast and 20 × 2.67 Gy to the tumour bed; five were treated with 15 × 2.67 Gy; and five received hypofractionated treatment with 5 × 5.2 Gy.

### Analysis of the breathing signal

The *amplitude per vmDIBH, calculated from* the 95 vmDIBHs (recorded during the CBCT immediately after the fraction) was on average 4.2 mm (range 0.7 – 11.9 mm) during the first and second vmDIBH, which was 1.2 mm larger than the predefined gating window (3 mm) (SD 1.9 mm, range −2.0 to 4.1 mm). The mean difference in vmDIBH level between the CT reference and the CBCT after the fraction was on average 2.0 mm (SD 2.5 mm, range −1.0 mm tot 8.8 mm). The average *duration of the BH* during the CBCT after the fraction was 30 s (SD 6.0 s). During the CBCTs, patients *maintained BH* within the 3.0 mm gating window in 39% of the BHs. 49% of the BHs would maintain within a 4.0 mm gating window.

### Recalculation of the 3D dose distribution

For 11 patients, three CBCTs were available, for one patient two CBCTs and for one patient only one CBCT was available, resulting in 36 CBCTs in total. In all patients treated with SIB (three patients with seven CBCTs), the coverage of the CTV2 fulfilled the predefined clinical specifications, with a mean V_95%_ of 100% (SD 0.02%). In seven of the remaining ten patients (15 of the remaining 29 CBCTs), the coverage of CTV1 did not fulfil the requirement of V_95%_ >99%; the V_95%_ was on average 98.4% (SD 1.7%; range 92.6–100%), and only one patient had in one vmDIBH a V_95%_ below 95%. The values of the MHD and MLD were all within the predefined specifications in all CBCTs (Table 2).

**Table 2:** Average values with range and SD for the V_95%_ of the clinical target volumes (CTV1 = elective volumes, CTV2 = boost volumes) and for the mean heart dose and the mean lung dose: planning criteria/constraints, values in the treatment plan based on the planning CT, and values based on the recalculated stitched CBCT. Delta values represent the difference between recalculated CBCT and planning CT. p-values are based on one-sample t-tests comparing recalculated CBCT values to planning CT values.

	**Planning-criteria/constraints**	**Planning CT N = 13**	**Recalculated based upon stitched CBCT N = 13 patients, 36 CBCTs**
**V95% CTV1** Δ = −0.9% p = 0.007	>99%	99.3 (98.6–99.9%) (SD 0.48%) (N = 10)	98.4% (92.6 – 100%) (SD 1.7%) (N = 10; 29 CBCTs)
**V95% CTV2** Δ = 0% p = 0.192	>99%	100% (100%) (SD 0%) (N = 3)	100% (100%) (SD 0%) (N = 3; 7 CBCTs)
**Mean Heart Dose** Δ = 0 Gy p = 0.637	<2.0 Gy w/o boost <3 Gy with boost	Gy (0.3–2.8 Gy) (SD 0.9 Gy)	Gy (0.1 – 2.8 Gy) (SD 0.9 Gy)
**Mean Lung Dose** Δ = +0.2 Gy p = 0.008	<3 Gy w/o boost <5 Gy with boost	2.7 Gy (0.6–5.1 Gy) (SD 1.5 Gy)	2.9 Gy (0.6–5.3 Gy) (SD 1.5 Gy)

Comparison of DVH parameters between the recalculated CBCTs and the corresponding planning CTs, showed very small differences: the mean difference in V_95%_-CTV1 was −0.8% (SD 1.6%, p = 0.007); the mean difference in V_95%_ −CTV2 was 0.0% (SD 0.0%, p = 0.192). The average difference in MHD and MLD was 0.0 Gy (SD = 0.0 Gy, p = 0.637), and 0.1 Gy (SD = 0.0 Gy, p = 0.007), respectively (Table 2).

### Correlation between differences in breathing signal and differences in DVH parameters

**Fig. 2:**
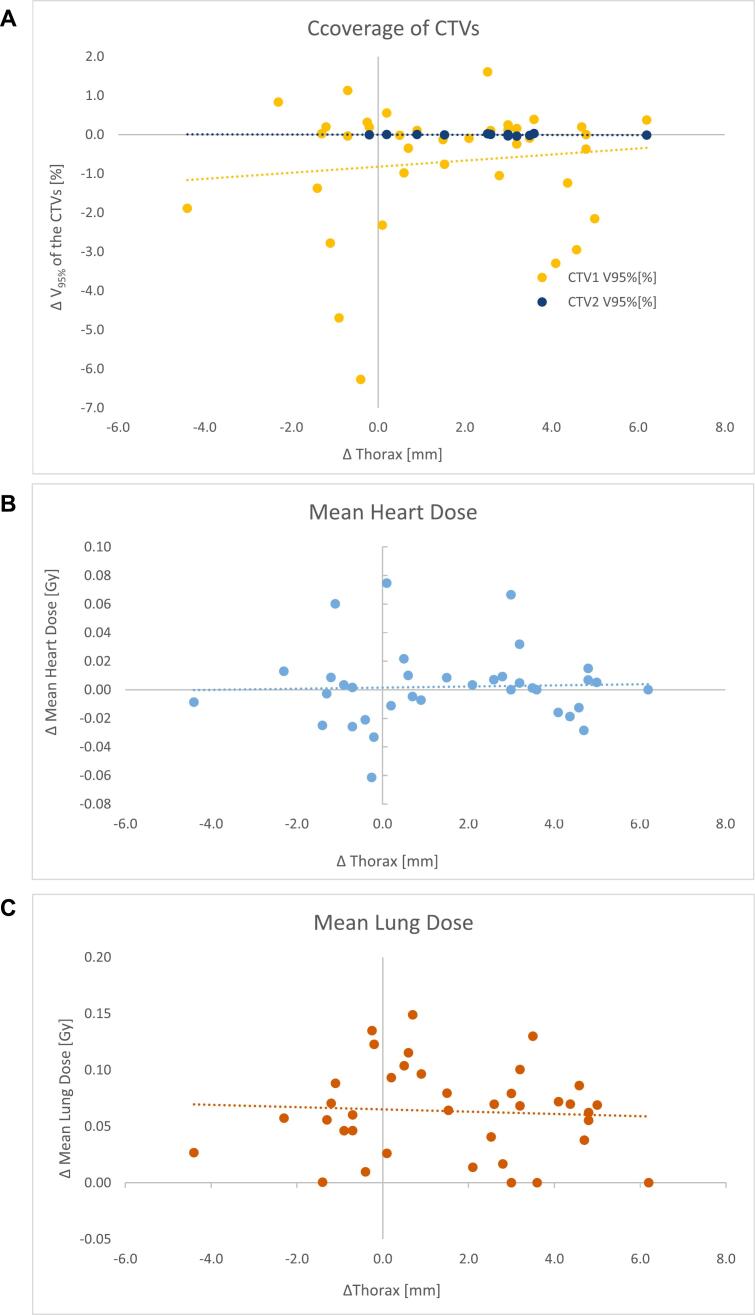
Correlation between the difference in breathing level (Δ Thorax) and the difference in the V_95%_ of the CTV1 and CTV2 (Fig. 2A), and the difference in the mean heart dose (MHD) (Fig. 2B), and mean lung dose (MLD) (Fig. 2C). The values of R^2^ for CTV1, CTV2 and the MHD were 0.0142, 0.0297, 0.0014, and 0.0039 respectively.

No significant correlation (p < 0.05) was found between the difference in breathing level and the difference in coverage of the CTV1 and CTV2 (Fig. 1A), and the MHD (Fig. 1B) and MLD (Fig. 1C). The V_95%_-CTV1 in patients with a vmDIBH moving out of the 3 mm window was on average −1.43% (range −6.27% − 0.39%) lower than at the planning CT, whereas this was only −0.16% (range −2.95% − 1.13%) lower for patients who maintained their vmDIBH within the 3 mm window (p = 0.016). For the delta MHD these values were 0.02 Gy (range −0.02 – 0.08 Gy) vs. −0.01 Gy (range −0.06 – 0.01 Gy), respectively (p = 0.012). No significant differences in the delta MLD were observed (p = 0.379) (Table 3A). In addition, no significant differences were seen in delta DVH values between patients with the vmDIBH central staying in or outside of the 3 mm window (Table 3B). (See Fig. 2A).

**Table 3:** Differences in Dose Volume Histogram parameters between the planning CT and the recalculated dose distributions on the stitched CBCT, for patients who maintained their vmDIBH (voluntary moderately deep inspiration breath hold) within or outside the predefined 3 mm window (Table 3A), and for patients with a breathing level (middle position) within or outside the 3 mm window (Table 3B). MHD = Mean Heart Dose; MLD = Mean Lung Dose.

**DVH parameter**	**Mean value (range, SD) of patients with a constant vmDIBH within the 3 mm window** **N = 19 CBCTs in 8 patients**	**Mean value (range) of patients with a vmDIBH moving outside the 3 mm window N = 17 CBCTs in 7 patients**	**p-value** **Mann Whitney-*U* test**
Delta V_95%_CTV1 between planning CT and the CBCT	−0.16% (−2.95–1.13%) (SD = 0.93%)	−1.43% (−6.27–––0.39%) (SD = 1.94%)	0.016
Delta MHD between planning CT and CBCT	−0.01 Gy (−0.06–0.01 Gy) (SD = 0.02 Gy)	0.02 Gy(−0.02–0.08 Gy) (SD = 0.028)	0.012
Delta MLD between planning CT and CBCT	0.07 Gy (0.00––0.14 Gy (SD = 0.05 Gy)	0.05 Gy (0.000–0.10 Gy) (SD = 0.03 Gy)	0.379

**DVH parameter**	**Mean value (range, SD) of patients with a middle position of the vmDIBH within the 3 mm window N =17 CBCTs in 7 patients**	**Mean value (range) of patients with a middle position of the vmDIBH outside the 3 mm window N = 19 CBCTs in 9 patients**	**p-value Mann Whitney-*U* test**
Delta V_95%_CTV1 between planning CT and the CBCT	−0.97% (−6.27–1.13%) (SD = 2.00)	−0.58% (−3.30–0.83%) (SD = 1.19%)	0.827
Delta MHD between planning CT and CBCT	0.00 Gy (−0.06–0.08 Gy) (SD = 0.03 Gy)	0.00 Gy (−0.03–0.07 Gy)(SD = 0.2 Gy)	0.452
Delta MLD between planning CT and CBCT	0.08 (0.00–0.15 Gy) (SD = 0.04 Gy)	0.05 Gy (0.00–0.13 Gy) (SD = 0.04)	0.093

## Discussion

To our knowledge, this is the first study where intra-and inter- breath-hold variations during vmDIBH were correlated with dose-volume parameters. We found that 61% of vmDIBHs performed during CBCT acquisition without visual coaching exceeded the predefined 3 mm gating window, as measured using the Catalyst surface tracking system. On average, the breathing level during the CBCT was 2.0 mm higher (SD = 2.5 mm) than during the CT-scan, and the amplitude was 3.6 mm, which is 0.6 mm (SD = 1.9 mm) larger than the 3 mm reference window. Despite these deviations, recalculated dose distributions showed that all tumour bed CTVs (CTV2) met clinical coverage criteria. For the whole breast CTV (CTV1), the V_95%_ was lower than the 99% threshold in 7 of 10 patients, but only one patient had a V_95%_ below 95%. All MHD and MLD values remained within clinical limits. A small systematic difference between breathing levels during planning CT and treatment may reflect patient acclimatization, this has to be investigated. Prior work shows that visual feedback can improve breath‑hold reproducibility compared with audio‑only coaching, which may partly relate to enhanced task control and confidence [25]. Practical implementation experiences also highlight benefits and operational considerations when deploying DIBH programs [35].

We assumed that staying within a 3 mm window would ensure acceptable dose distributions. Although amplitudes during CBCT were larger than expected (mean 4.2 mm), dosimetric deviations remained minimal and consistent with other findings [24].

Although some differences in CTV coverage and MHD reached statistical significance between patients within and outside the 3 mm window, these were small and not clinically relevant. Minor vmDIBH level deviations did not substantially affect dose delivery, suggesting continuous surface monitoring may not be essential for all patients. DVH differences between planning CT and CBCTs were minimal. While our planning goal was V95% > 99% for the PTV, a V95% > 95% for the CTV in a single fraction is often considered acceptable [36]. The still good coverage seen in our patients, despite breathing motion outside the 3.0 mm window is likely due to the robustness of our hybrid technique with ∼ 80% dose from tangential fields [37]. Only one vmDIBH dropped below the 95% threshold (92.6% of the prescribed dose). However, even in this worst case, this did not affect the OAR constraints. Quantitatively, only 1/150 vmDIBH manoeuvres (0.7%), in 1/13 patients, was below V95% = 95%, with all MHD/MLD values within predefined limits.

In a small convenience survey (n = 20), 75% of patients indicated a preference for audio‑visual feedback during vmDIBH. While our dosimetric results suggest audio‑only coaching was sufficient under the tested conditions, audio‑visual feedback may still be selectively offered for patient comfort or in settings prioritising very tight reproducibility [25,35]. This study has several strengths. We were able to correlate breathing motion with DVH parameters using multiple measurements per patient. However, several limitations must be acknowledged. First of all, we had a limited sample size: only 13 patients had complete CBCT data, reducing statistical power. In addition, breathing motion was only assessed during CBCT acquisition, which may not reflect variability during the full treatment delivery. The recalculation using CBCTs stitched to the planning CT may have some geometric and dose-accuracy uncertainties, since CBCTs are known to underestimate Hounsfield Units, particularly in lung tissue [38]. However, we expect that these effects were so small that they are not considered clinically relevant. It should also be noted that the analysis was limited to the short duration of the CBCT acquisition, which may not fully represent the longer treatment delivery period. Finally, the Catalyst system did not allow automatic storage of breathing signals during CBCT, requiring manual video capture, which may have limited the accuracy of the analysis. Despite these limitations, our findings support the feasibility of vmDIBH without visual feedback in most patients. Further research is needed to assess whether these results hold for other planning techniques, such as IMRT, and over longer treatment durations.

## Conclusion

In conclusion, this is the first study to relate intra‑ and inter‑fraction vmDIBH variations to 3D dose using CBCT-based recalculation. Although vmDIBH motion frequently exceeded the 3 mm window, the dosimetric impact was minimal: only 1 out of 150 vmDIBH manoeuvres (0.7%), in 1 of 13 patients, fell below V95% = 95%, while all mean heart and lung doses remained within clinical limits. Under our tested conditions, vmDIBH without visual coaching yielded clinically acceptable target coverage and OAR doses, suggesting no direct need for visual feedback beyond potential comfort benefits. External validation in other techniques and workflows remains warranted.

## Declaration of competing interest

The authors declare that they have no known competing financial interests or personal relationships that could have appeared to influence the work reported in this paper.
